# A model of porcine polymicrobial septic shock

**DOI:** 10.1186/s40635-023-00513-7

**Published:** 2023-06-02

**Authors:** Finnja Marie Zurek-Leffers, Florian Lehmann, Laura Brabenec, Sebastian Kintrup, Katharina E. M. Hellenthal, Kira Mersjann, Felicia Kneifel, Michael Hessler, Philip-Helge Arnemann, Tim-Gerald Kampmeier, Christian Ertmer, Patrick Kellner, Nana-Maria Wagner

**Affiliations:** 1 Department for Anaesthesiology, Intensive Care and Pain Medicine, University Hospital Münster, Albert-Schweitzer-Campus 1, 48149 Münster, Germany; 2 Department of General, Visceral and Transplant Surgery, University Hospital Münster, Münster, Germany; 3 Department of Anaesthesiology and Intensive Care Medicine, University Medical Center Schleswig-Holstein, Campus Lübeck, Lübeck, Germany

**Keywords:** Sepsis, Septic shock, Pig, Animal model, Faecal peritonitis

## Abstract

**Background:**

Sepsis is a life-threatening organ dysfunction caused by a dysregulated host response to infection. Mortality of patients with sepsis is high and largely unchanged throughout the past decades. Animal models have been widely used for the study of sepsis and septic shock, but translation into effective treatment regimes in the clinic have mostly failed. Pigs are considered as suitable research models for human diseases due to their high comparability and similarity to human anatomy, genetics, and the immune system. We here evaluated the previously reported models of septic shock in pigs and established a novel model of polymicrobial sepsis that meets the clinical criteria of septic shock in pigs.

**Materials and methods:**

The literature search was performed using the keywords “pig”, “sepsis” and “septic shock”. For the establishment of septic shock in *n* = 10 German landrace pigs, mechanical ventilation was initiated, central venous and arterial lines and invasive hemodynamic monitoring via pulse contour cardiac output measurement (PiCCO) established. Peritoneal polymicrobial faecal sepsis was induced by application of 3 g/kg body weight faeces into the abdominal cavity. Septic shock was defined according to the third international consensus definitions (Sepsis-3). Upon shock, pigs underwent the 1-h bundle for the treatment of human sepsis. Cytokine levels were measured by ELISA.

**Results:**

Published porcine sepsis models exhibited high methodological variability and did not meet the clinical criteria of septic shock. In our model, septic shock developed after an average of 4.8 ± 0.29 h and was associated with a reproducible drop in blood pressure (mean arterial pressure 54 ± 1 mmHg) and significant hyperlactatemia (3.76 ± 0.65 mmol/L). Septic shock was associated with elevated levels of interleukin-6 (IL6) and initial cardiac depression followed by a hyperdynamic phase with significant loss of systemic vascular resistance index after initial resuscitation. In addition, organ dysfunction (acute kidney injury) occurred.

**Conclusions:**

We here established a model of septic shock in pigs that meets the clinical criteria of septic shock utilized in human patients. Our model may thus serve as a reference for clinically relevant sepsis research in pigs.

**Supplementary Information:**

The online version contains supplementary material available at 10.1186/s40635-023-00513-7.

## Background

Sepsis is a life-threatening disease defined by an organ dysfunction caused by a dysregulated host response to infection. Sepsis and septic shock are major healthcare problems and have a high impact on morbidity and mortality worldwide. In 2016 the third international consensus definitions of sepsis and septic shock (Sepsis-3) were published, resulting in a new definition that focuses on organ dysfunction and the systemic inflammatory host response [34]. Septic shock, defined as sepsis with mean arterial pressure (MAP) below 65 mmHg despite adequate volume therapy and serum lactate above 2 mmol/L, is associated with higher mortality [7, 10, 20, 34]. Early and goal directed therapy can improve the outcome in septic patients [7, 10]. However, even after decades of research, a therapy that successfully addresses the hosts dysregulated, overshooting immune response is unavailable. Therefore, studies on the pathophysiology of sepsis as well as septic shock and potential therapeutic targets are required. Due to the complexity of this disease, in vitro protocols are unable to reproduce the full spectrum of the host response. Thus, animal models are needed to replicate the complex pathophysiology of sepsis and its treatment options [12, 18]. However, there remains a large discrepancy between animal models and human septic patients in intensive care units (ICU) shown by the inadequate efficacy of treatments that are considered beneficial in animal models but not in humans [11].

Several limitations apply to animal models used for the evaluation of therapeutic strategies for the treatment of sepsis [11, 18]. For example, models vary in which sepsis or endotoxemia are induced in different ways such as cecal ligation and puncture or the administration of lipopolysaccharide or live bacteria [8, 18, 30]. In addition, these models are commonly used in young and healthy animals without any comorbidities, thus lacking clinical comparability with the usually elderly patients in the ICU [11]. Moreover, animal sepsis models often lack clear criteria for defining sepsis and septic shock. Therefore, their translation to humans is often limited. An animal that is highly comparable to humans in terms of anatomy, genetics and physiology is the pig. A crucial point in the study of infectious diseases in porcine models is that pigs possess a variety of innate and acquired immune effectors that are structurally and functionally comparable to those in humans and—importantly—more similar to humans than mice [21, 24, 28]. However, the existing models are heterogeneous in terms of their adherence to clinically established standards and exhibit reproducibility. Nevertheless, these points are necessary for optimal research conditions. They require stable sepsis models that are both reproducible and comparable in terms of disease severity and clinically relevant. Despite all limitations, animal models remain the gold standard for the study of complex diseases such as sepsis [8].

We here first systematically evaluated the literature for clinically relevant models of porcine sepsis. Based on the heterogeneity of our results, our aim was then to develop a porcine sepsis model that meets the clinical criteria of septic shock according to the third international consensus definitions (Sepsis-3) [7, 29, 34]. The goal of this study was to develop a protocol of porcine sepsis that is highly reproducible, enables the comparison of results and is of robust translational relevance for human sepsis research.

## Materials and methods

### Literature research

The aim of this study was to develop a model of septic shock in pig that can provide a realistic and clinically relevant starting point for further investigations. To provide a feasible basis for further research, the model must be implementable within a realistic time. In our specific case, this means that septic shock, defined by clinical criteria, should occur within a few hours to provide a meaningful starting point for further therapies. Using the keywords "sepsis AND septic shock AND pig", a systematic literature search was performed in the PubMed® database. 305 entries from the years 2002–2022 were evaluated. The obtained publications were examined for the applicability of the criteria of septic shock in humans (i.e., mean arterial pressure of less than 65 mmHg and elevated levels of serum lactate of more than 2 mmol/L [34]) together with the type and mode of sepsis induction and the age/weight as well as the sex of the used animals was analysed. Considering the above mentioned quality criteria for a model of septic shock in pigs, a cutoff for the occurrence of shock defined as a serum lactate level > 2 mmol/L and a mean arterial pressure < 65 mmHg within 8 h after sepsis induction was chosen. The corresponding pig models were limited to in vivo models of polymicrobial sepsis with septic shock. Publications that used a two-hit model (e.g., hemorrhage/ischemia and sepsis) or neonatal pigs were excluded. Publications (*n* = 7) without data about MAP and/or lactate levels were also excluded from Table 1. References of Table 1 are listed in Additional file 1: Table S1.

**Table 1:** Systematic literature review of porcine septic shock models used from the years 2002–2022 and their characteristics

Author	MAP < 65 mmHg	Lactate ≥ 2 mmol/L	Weight (kg)	Sex	Sepsis induction (faecal amount)
Marx et al., 2002	Yes	No	20.8 ± 1.8	e	1 g/kg i.p.
Hiltebrand et al., 2003	Yes	No	20–25	–	20 g i.p.
Krejci et al., 2003	Yes	No	22–28	–	20 g i.p.
Hiltebrand et al., 2004	Yes	No	20–25	–	20 g i.p.
Marx et al., 2004	Yes	No	23.1 ± 2.4	f	0.75 g/kg i.p.
Marx et al., 2006	No	No	22.9 ± 2.8	f	0.75 g/kg i.p.
Krejci et al., 2006	Yes	No	22–28	–	20 g i.p.
Schuerholz et al., 2006	No	No	21.2 ± 1.5	–	0.75 g/kg i.p.
Azevedo et al., 2007	Yes	No	40–45	–	1.5 g/kg i.p.
Hiltebrand et al., 2007	No	No	28–32	–	20 g i.p.
Krejci et al., 2007	No	No	28–32	–	3 g/kg i.p.
Barth et al., 2008	No	No	–	e	Faeces i.p.
Hauser et al., 2009	No	No	46–59	e	0.05 g/kg i.p.
Simon et al., 2009	No	No	–	–	1 g/kg i.p.
Sykora et al., 2009	Yes	No	32–38	e	0.5 g/kg i.p.
Wauters et al., 2010	No	No	40.5 ± 4	f	0.8 g/kg i.p.
Rosário et al., 2011	Yes	No	35–45	–	0,75 g/kg i.p.
Kuncová et al., 2011	No	No	–	e	0.5 g/kg i.p.
Regueira et al., 2012	No	No	36–46	e	1 g/kg i.p.
Ji et al., 2012	Yes	No	26–35	f	1 g/kg i.p.
Derive et al., 2013	No	No	30–40*	m	1.5 g/kg i.p.
Kieslichova et al., 2013	Yes	No	36*	–	CLP 3 cm
Li et al., 2013	Yes	No	31 ± 5	m	0.5 g/kg i.p.
Schmidt et al., 2013	Yes	No	29.8 ± 3.5	f	0.75 g/kg i.p.
Corrêa et al., 2014	Yes	No	40–43	m	2 g/kg i.p.
Jarkovska et al., 2015	Yes	No	39 ± 6	e	1 g/kg i.p.
Nußbaum et al., 2016	No	No	65–73	m	1 g/kg dissolved in 500 mL, 3 mL/kg i.p.
Corrêa et al., 2017	No	No	–	–	1–2 g/kg i.p.
Nußbaum et al., 2017	No	No	55–62	m	1 g/kg i.p.
Ospina-Tascón et al., 2017	Yes	No	35–42	f	1.5 g/kg i.p.
Laroye et al., 2018	No	No	40–60	m	3 g/kg i.p.
Jarkovska et al., 2018	Yes	No	43.9 ± 5.8	e	1 g/kg i.p.
Ferrario et al., 2019	Yes	No	42.3 ± 3.9	e	3 g/kg i.p.
Kurtz et al., 2019	No	No	40	m	0.5 g/kg i.p.
Carrara et al., 2019	Yes	No	43.8 ± 3.9	e	3 g/kg i.p.
Park et al., 2019	Yes	No	49 ± 8	m	1 g/kg i.p.
Chvojka et al., 2020	Yes	No	54	e	2 g/kg i.p.
Horak et al., 2020	Yes	No	40–46	e	1 g/kg i.p.
Merz et al., 2020	Yes	No	65–73	m	Faeces i.p.
Al-Obeidallah et al., 2021	Yes	No	40 ± 6	e	1 g/kg i.p.
Bollen Pinto et al., 2022	Yes	No	42.3 ± 3.9	e	3 g/kg i.p.
Garcia et al., 2022	Yes	No	49 ± 5	e	3 g/kg i.p.
Messerer et al., 2022	No	No	54–89	e	1.5 g/kg i.p.
Rutai et al., 2022	Yes	Yes	35 ± 9*	e	0.6 g/kg i.p.

Criteria for: hypotension: Yes = MAP < 65 mmHg or catecholamine requirement for MAP > 65 mmHg. Hyperlactatemia: Yes = lactate > 2 mmol/L within 8 h after sepsis induction, all animals with hyperlactatemia, baseline values < 2 mmol/L. Weight: * = mini-pig. Sex: – = not mentioned, f = female, m = male, e = either. i.p. = intraperitoneal, CLP = cecal ligation and puncture. Complete list of references in the Additional file 1: Table S1

### Animals and animal preparation

The present study was approved by the Animal Care Committee of the State Government of North-Rhine Westphalia (LANUV NRW, Recklinghausen, Germany; Approval AZ 81-02.04.2020.A428). All methods were performed in accordance with the National Institutes of Health Guide and as well as the American Physiologic Society’s “Guide for the Care and Use of Laboratory Animals”.

After an adaptation period of 1 week and a withdrawal of food for 12 h with water ad libitum, 10 healthy female German landrace pigs with a body weight of 29.5 ± 4.4 kg were anaesthetized by intramuscular injection of S-ketamine (15 mg/kg, Pfizer Inc, New York, USA) and azaperone (2 mg/kg, Elanco Animal Health Inc, Bad Homburg, Germany). Subsequently, venous access (20G) was established into an ear vein. After deepening anaesthesia with intravenous administration of propofol (2–3 mg/kg, CP-Pharma Handelsgesellschaft mbH, Burgdorf, Germany), the pigs were endotracheally intubated with an ID 7.5 tube (Teleflex Medical Europe Ltd, Athlone, Ireland) to provide mechanical ventilation (Cato®, Dräger Medical Deutschland GmbH, Lübeck, Germany) with a tidal volume of 7–10 mL/kg adjusted to an expiratory carbon dioxide partial pressure of 40 ± 5 mmHg and an inspiratory oxygen fraction of 30 ± 5%. Anaesthesia was continued with isoflurane (1.2–1.4Vol% et, Isofluran vet., Baxter Deutschland GmbH, Unterschleißheim, Germany) and continuous analgesia was provided by intravenous fentanyl (0.005 mg/kg/h, Janssen-Cilag GmbH, Neuss, Germany). Anaesthesia was maintained until the end of the study. All subsequent catheterizations and surgical procedures were performed under sterile conditions and after ensuring adequate general anaesthesia. After ultrasound-guided placement of a quad-lumen central venous catheter (Teleflex Medical Europe Ltd, Athlone, Ireland) in the jugular vein using Seldinger’s technique and a 13F high-flow catheter (HF-TLK, Achim Schulz-Lauterbach VMP GmbH, Iserlohn, Germany) in the femoral vein, a pulse contour cardiac output catheter was placed in the left femoral artery (PULSION Medical Systems AG, Munich, Germany). The intravascular catheters were sewn on and connected to a transpulmonary thermodilution and pulse contour cardiac output computer (PiCCO2, PULSION Medical Systems AG, Munich, Germany) for continuous analysis of hemodynamic parameters.

### Experimental protocol

**Fig. 1:**
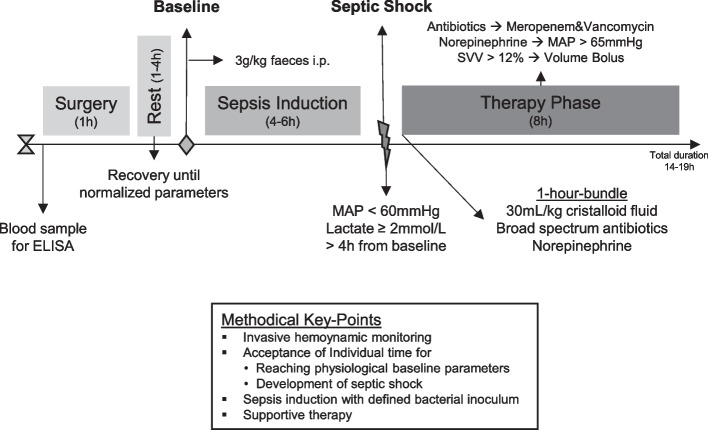
Timeline of the experimental protocol. After surgery and recovery time until hemodynamic and laboratory parameters were in a physiological range, baseline measurements were performed. Septic shock (defined according to the Sepsis-3 criteria) was induced by intraperitoneal injection of 3 g/kg BW feces. At the onset of septic shock (mean 4.8 ± 0.29 h post sepsis induction), a therapy phase over 8 h was succeeded according to the sepsis guideline with application of the 1 h-bundle, followed by a differentiated hemodynamic as well as volume therapy

After placement of intravascular catheters, a median laparotomy was performed. An electrocautery (ERBE VIO 300 D, ERBE Elektromedizin GmbH, Tübingen, Germany) was used immediately after skin incision to stop minimal bleeding from dermal and muscular vessels. An abdominal drain (Ch25) was placed between the small intestinal loops to exclude encapsulation or merely localized peritonitis following faeces application and a 12Fr balloon catheter (Teleflex Medical Europe Ltd, Athlone, Ireland) was surgically inserted into the urinary bladder to measure urine output. After placement of the drainages, the abdominal cavity was surgically closed. During surgery, the animals received a fluid bolus of crystalloid fluid (Sterofundin ISO, B. Braun SE, Melsungen, Germany, 10 mL/kg) in addition to the basic fluid requirement, which was met by a continuous infusion of 2 mL/kg/h during the experiment. After instrumentation and surgery, the pigs were allowed to recover until hemodynamic and laboratory parameters normalized for at least 1 h. At this timepoint baseline measurements were performed and sepsis was induced by injecting 3 g/kg bodyweight (BW) of faeces, collected the day before and incubated overnight (for at least 19 h) at 37 °C in 200 mL 1% glucose solution, through the drainage without prior filtration into the abdominal cavity. The occurrence of septic shock was defined as MAP < 60 mmHg in combination with serum lactate ≥ 2 mmol/L at a timepoint at least 4 h apart from faeces injection. During the establishment phase of the model, preliminary experiments showed that 1.5 g/kg faeces did not sufficiently induce septic shock. If criteria for septic shock were met a second set of measurements was taken. Afterwards treatment was initiated according to the 1-h-bundle of the surviving sepsis campaign including an i.v. fluid bolus (Sterofundin ISO, B. Braun SE, Melsungen, Germany) of 30 mL/kg over 1 h. In addition, broad spectrum antibiotic therapy (meropenem (Fresenius Kabi Deutschland GmbH, Bad Homburg, Germany) bolus 20 mg/kg, followed by continuous infusion (2 mg/kg/h) and vancomycin (Hikma Pharmaceuticals PLC, London, United Kindom) bolus 20 mg/kg over 1 h followed by continuous infusion (0.5 mg/kg/h)) was administered for the rest of the experiment. Norepinephrine (Sanofi-Aventis Deutschland GmbH, Frankfurt, Germany) was administered continuously and titrated to maintain a MAP > 65 mmHg. The treatment protocol was continued for 8 h after the onset of septic shock. After completion of the initial fluid bolus the pigs received additional fluid boluses of 5 mL/kg over 15 min if stroke volume variation (SVV) exceeded 12% (Fig. 1). If hypoglycaemia occured (serum glucose < 70 mg/dL), glucose was substituted using 40% glucose solution (Glucose40%, B. Braun SE, Melsungen, Germany).

### Measurements and blood sampling

Hemodynamic parameters were observed continuously and extended hemodynamic parameters were measured hourly by thermodilution. The body surface area was calculated according to Kelley et al. (1973) [17]. Urine output and ventilatory parameters were also documented hourly. Blood sampling was performed hourly by bedside arterial and central venous blood gas analysis, including sodium, potassium, calcium, glucose, lactate and creatinine (epoc® Blood Analysis Systems with epoc® BGEM3 test cards, Siemens Healthcare GmbH, Erlangen, Germany). For immunoassay analysis, EDTA-stabilized blood was drawn preoperatively and at shock time, centrifuged, and the collected plasma fraction was frozen at − 80 °C until analysis. SVV and fluid bolus administration were documented every 15 min during the treatment phase.

### Proinflammatory mediators and glycocalyx marker

For the determination of proinflammatory mediators and their changes during the development of septic shock, enzyme-linked immunosorbent assay (ELISA) (Cytokine and Chemokine 9-Plex Porcine ProcartaPlex™ Panel 1, ThermoFisher Scientific Inc., Waltham, Massachusetts, USA) was used following the instructor’s guide for *n* = 6 pigs preoperatively and at time of shock. To analyze glycocalyx damage, syndecan-1 ELISA (Pig Syndecan 1 (SDC1) ELISA Kit, Abbexa LTD, Cambridge, UK) was performed for *n* = 10 animals at the beginning of the experiment and at the end of the experiment, 8 h after the onset of septic shock (S + 8) according to the manufacturer's instructions.

After the end of the protocol pigs were euthanized with 200 mL potassium chloride (KCL) 7.46% (Deltamedica GmbH, Reutlingen, Germany) under deep anaesthesia and tissue samples were collected*.*

### Microbiological and histopathological analysis

To differentiate the bacterial inoculum, faeces samples (*n* = 6) were analysed microbiologically and evaluated in terms of bacterial species and bacterial counts (colony forming units (CFU)). Bacteria were grown on blood agar (total CFU) (BD Columbia 5% SB/COL-S, BD, Heidelberg, Germany), MacConkey (BD Mac Conkey II, BD, Heidelberg, Germany) and CNA agar (BD CNA Improved II, BD, Heidelberg, Germany) for 24 h at 37 °C, and bacterial species were analysed by Matrix Assisted Laser Desorption Ionization Time of Flight (MALDI Biotyper Sirius One, Bruker Corporation, USA).

The porcine kidney biopsies from the medulla of *n* = 10 septic pigs and *n* = 5 healthy controls, kept in 4% formaldehyde at room temperature, were embedded using Microm STP 120 Spin Tissue Processor (Thermo Fisher Scientific, Germany) following coating with paraffin (Thermo Fisher Scientific, Germany) at 60 °C. Next, 6 μm thick slices were made using Microm HM 355 S (Thermo Fisher Scientific, USA), then transferred to a microscope slide (Thermo Scientific, Germany) and put on a slide warmer (Adamas Instrumenten, Netherlands) at 37 °C to dry. After incubation at 37 °C over night, the histology cross sections were stained in hematoxylin–eosin (HE) (Merck, Germany). Representative pictures were taken with a LionHeart FX Automated Microscope (BioTek Instruments, USA, Software Gen5 Version 3.05). In histopathology, acute kidney injury is defined as “acute tubular injury” (ATI) [14]. Thus, tubular injury was quantified by a score established by Rong et al.: presence or absence of epithelial necrosis, loss of brush border, cast formation, tubular dilatation and classified on a five-point-scale: 0: healthy kidney; 1: 1–25%; 2: 26–50%; 3: 51–75%; and 4: 76–100% tubuli affected [31]. In addition, glomerular integrity [13] was quantified in low power fields as follows: 1: 1–25%; 2: 25–50%; 3: 50–75%; and 4: 75–100% collapsed glomeruli.

### Statistics

For statistical analysis GraphPad Prism 7 software (GraphPad Software Inc., San Diego, California, USA) was used. To test for normal distribution the D’Agostino&Pearson as well as the Shapiro–Wilk normality test were used. Statistics were performed using a paired *t* test or a Wilcoxon matched-pairs signed rank test/Mann–Whitney test if the data were not normally distributed. When multiple measurement timepoints were compared, a repeated measures (rm) ANOVA with Geisser–Greenhouse correction and Bonferroni's multiple comparisons test was used. A *P* value of less than 0.05 was considered statistically significant. Data are represented as mean ± SEM or boxplot (Tukey).

## Results

### Literature search

Of the 305 publications that were retrieved from MedLine by systematic literature search, 44 met the inclusion criteria (Table 1). Evaluation of the included publications revealed high methodological variability and mostly unmet obtainment of clinical criteria of septic shock. The criteria for septic shock (mean arterial pressure (MAP) < 65 mmHg and lactate ≥ 2 mmol/L) as well as the methodology for the induction of sepsis and septic shock in the examined models are listed in Table 1. The models exhibited extensive heterogeneity in methodology of sepsis induction from monomicrobial or polymicrobial infection to sole endotoxemia, whereby monomicrobial and endotoxemia models have not been included in Table 1. In addition, existing models often lack signs of hypoperfusion with elevated lactate levels or hemodynamic parameters that define septic shock but are not consistent with the Sepsis-3 definition [23, 35, 37]. Simultaneous occurrence of hypotension and hypoperfusion is also largely absent in published porcine models [1, 4, 23]. Other studies investigating septic shock do not provide clear definition criteria for the model [19, 26].

### Occurrence of septic shock

**Fig. 2:**
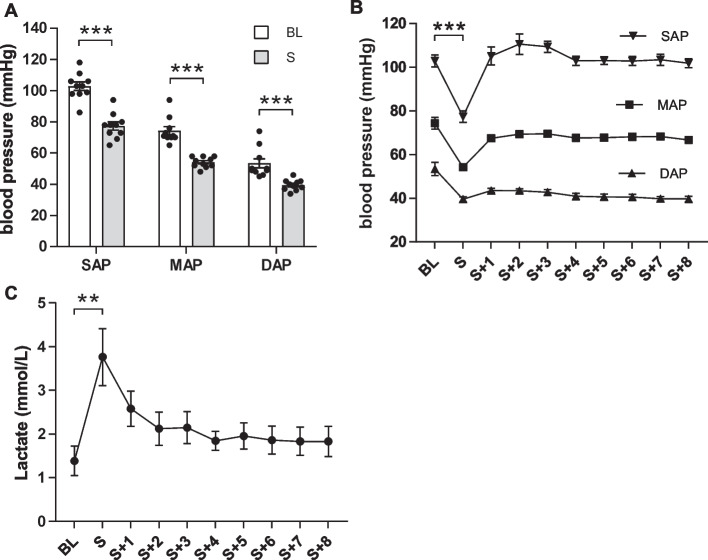
Occurrence of septic shock. **A** Significant drop in blood pressure at the time of septic shock, which here requires a MAP < 60 mmHg. MAP (BL) 74 ± 3 mmHg to MAP (S) 54 ± 1 mmHg. *n* = 10, Wilcoxon matched-pairs signed rank test. **B** Blood pressure during treatment period with a MAP goal ≥ 65 mmHg. *n* = 10. **C** Significant increase in serum lactate concentration from baseline (1.38 ± 0.34 mmol/L) to shock time (3.76 ± 0.65 mmol/L) with persistent hyperlactatemia after implementation of the 1 h-bundle (S + 1 2.58 ± 0.40 mmol/L) and over the treatment period. *n* = 10, rm ANOVA/Geisser–Greenhouse/Bonferroni. **P < 0.01, ***P < 0.001. BL = baseline, S = shock. mean ± SEM

In this large animal model, the onset of septic shock (S) (MAP < 60 mmHg and lactate > 2 mmol/L) was detected after an average of 4.8 ± 0.29 h after sepsis induction with a significant decrease in blood pressure to MAP 54 ± 1 mmHg compared to baseline values (BL, timepoint before faecal injection) (MAP 74 ± 3 mmHg; *P* < 0.001) (Fig. 2A, B). To ensure a definite and severe arterial hypotension in response to infection, we defined onset of septic shock by reaching MAP < 60 mmHg (instead of 65 mmHg). All animals required norepinephrine therapy to maintain MAP ≥ 65 mmHg despite fluid therapy. Significant hyperlactatemia with a mean serum lactate of 3.76 ± 0.65 mmol/L was detected at the time of septic shock, a significant increase from baseline serum lactate (1.38 ± 0.34 mmol/L; *P* = 0.009). During the treatment phase and after application of the 1 h-bundle, the hyperlactatemia declined (S + 1 h 2.58 ± 0.40 mmol/L) but did not reach baseline levels (Fig. 2C). Taken together, the clinical criteria of septic shock were met in all animals.

### Invasive hemodynamic measurements

**Fig. 3:**
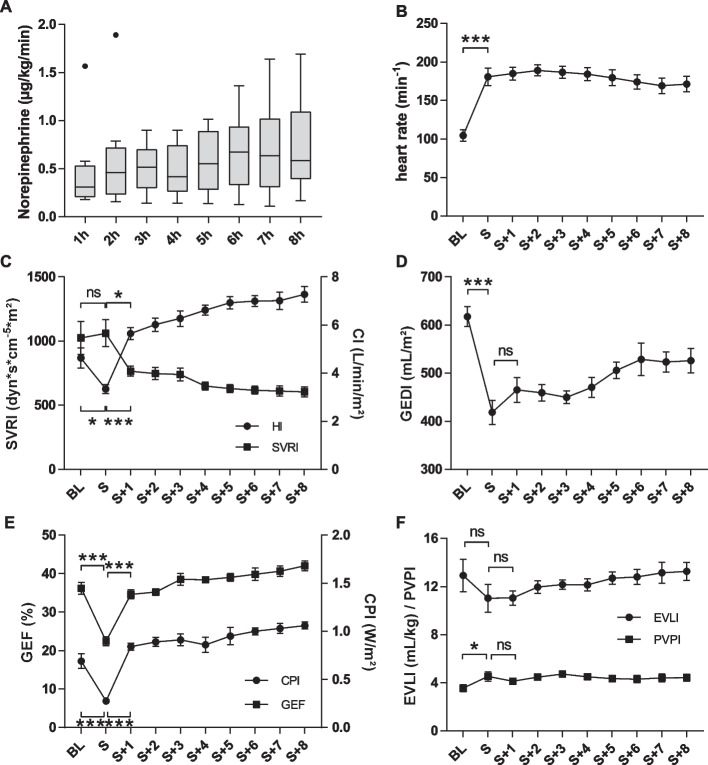
Changes in basic and extended hemodynamic parameters measured via thermodilution. **A** Vasopressor requirement middled per hour during the experiment with an interindividual variation and increase over the treatment period. *n* = 9. Boxplot (Tukey). **B** Significant increase of heart rate by onset of septic shock from BL (105 ± 7 min^−1^) to shock (181 ± 11 min^−1^). *n* = 10, paired *t* test. **C** Significant decrease in cardiac index in septic shock and significant increase after beginning of therapy. *n* = 10. No significant changes in SVRI at shock time; but significant loss of SVRI after first hour of therapy. *n* = 10, rm ANOVA/Geisser–Greenhouse/Bonferroni. **D** Significant decrease in GEDI by onset of septic shock (BL 681 ± 21 mL/m^2^; S 418 ± 25 mL/m^2^) without significant increase after implementation of the 1 h-bundle. *n* = 10, rm ANOVA/Geisser–Greenhouse/Bonferroni. **E** Cardiac output significantly decreased in septic shock and increased with start of therapy shown by changes in CPI as well as the changes in GEF. *n* = 10, rm ANOVA/Geisser–Greenhouse/Bonferroni. **F** Changes in pulmonary parameters (EVLI and PVPI) not significant; ns. *n* = 10, rm ANOVA/Geisser–Greenhouse/Bonferroni. *ns* not significant, *P < 0.05, ***P < 0.001. BL = baseline, S = shock. mean ± SEM

In the subsequent treatment phase, we frequently observed hemodynamic instability as evidenced by escalating norepinephrine dose during the experiment despite adequate and high-volume therapy. In addition, we observed interindividual differences in vasopressor requirement (Fig. 3A). Heart rate increased significantly in all animals from baseline 105 ± 7  to 181 ± 11 min^−1^ (*P* < 0.001) (Fig. 3B). At the timepoint of untreated shock we detected a significant decrease in cardiac output from baseline (cardiac index (CI): 4.63 ± 0.42 to 3.34 ± 0.18 L/min/m^2^, *P* = 0.016; cardiac power index (CPI): 0.69 ± 0.08 to 0.28 ± 0.03 W/m^2^, *P* = 0.019; global ejection fraction (GEF): 36.2 ± 1.5 to 22.4 ± 1.2%, *P* < 0.001) based on significantly lower preload, which was reflected by a significant change in global end-diastolic volume index (GEDI) (618 ± 21 to 418 ± 25 mL/m^2^, *P* < 0.001). After initial resuscitation, pigs developed a hyperdynamic circulation with a significant increase in CI and drop of systemic vascular resistance index (SVRI) (CI 3.35 ± 0.18 to 6.06 ± 0.27 L/min/m^2^, *P* < 0.001; CPI: 0.28 ± 0.03 to 0.9 ± 0.05 W/m^2^, *P* < 0.001; GEF: 22.4 ± 1.2 to 35.2 ± 1.7%, *P* < 0.001; SVRI: 1061 ± 105 to 766 ± 39 dyn*s*cm^−5^*m^2^, *P* = 0.009, Fig. 3C–E). Pulmonary edema parameters (extravascular lungwater index (EVLI) and pulmonary vascular permeability index (PVPI)) showed a significant increase in PVPI by shock time (BL: 3.55 ± 0.29 to S: 4.52 ± 0.38; *P* = 0.037) without significant changes in EVLI (Fig. 3F).

### Fluid resuscitation

**Fig. 4:**
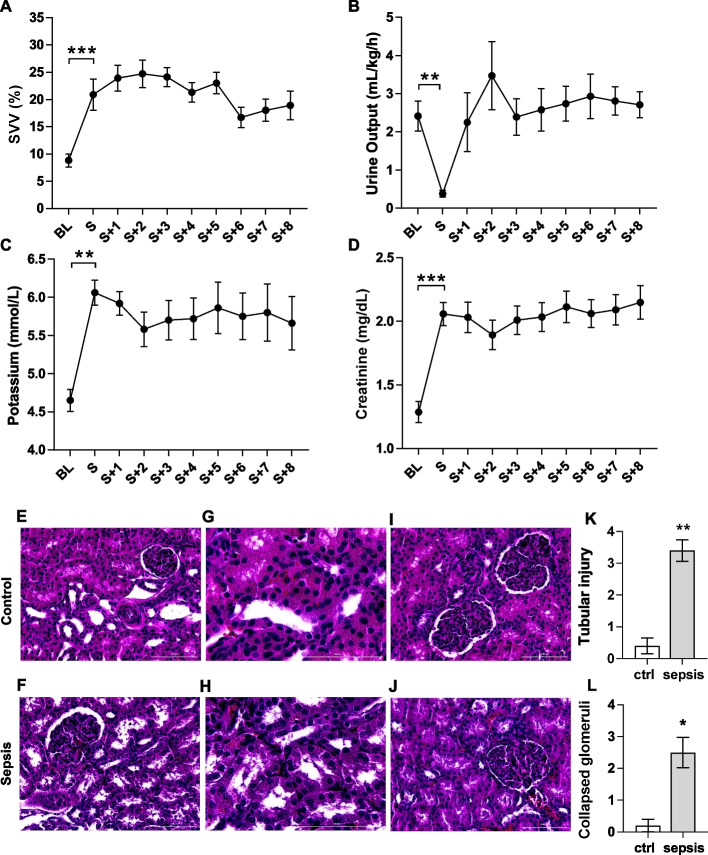
Emergence of acute kidney injury and volume requirement. **A** Volume need was observed by significantly increase of the SVV from baseline (8.8 ± 1.2%) to shock (22.7 ± 2.7%). *n* = 10, paired *t* test. **B** Onset of acute kidney injury shown by significant decrease of urine output (BL 2.4 ± 0.39 to S 0.18 ± 0.56 mL/kg). *n* = 10, Wilcoxon matched-pairs signed rank test. **C** AKI was accompanied by significant hyperkalaemia (BL 4.7 ± 0.14 mmol/L, S 6.1 ± 0.16 mmol/L). *n* = 10, Wilcoxon matched-pairs signed rank test. **D** Significant increase of creatinine from baseline (1.29 ± 0.08 mg/dL) to shock (2.06 ± 0.09 mg/dL), meeting the KDIGO criteria of AKI. *n* = 10, paired *t* test. mean ± SEM. **E**–**J** Histopathological changes in hematoxylin–eosin-stained kidney sections, top: control, below: sepsis. Scale bars indicate 100 µm. **E**–**H** Representative micrographs of tubular integrity. **I**–**J** Representative micrographs of glomerular integrity. **K** Quantitative summary of acute tubular injury in HE-stained kidney sections in sepsis (score 3.4 ± 0.3) with significant increase compared to healthy control (score 0.4 ± 0.2). *n* = 10 sepsis, *n* = 5 control, Mann–Whitney test. **L** Quantitative summary of collapsed glomeruli in HE-stained kidney sections with significant impaired glomerular integrity in 25–50% of the glomeruli in sepsis (score 2.5 ± 0.5) compared to healthy control (score 0.2 ± 0.2). *n* = 10 sepsis, *n* = 5 control, Mann–Whitney test. *P < 0.05, **P < 0.01, ***P < 0.001. BL = baseline, S = shock

According to the individualized fluid resuscitation protocol, animals received an average of 8130 ± 479 mL of crystalloid fluid throughout the experiment. All pigs showed significant haemoconcentration at the timepoint of untreated shock with an increase of haemoglobin (Hb) from 7.2 ± 0.2 g/dL at BL to 12.5 ± 0.4 g/dL (*P* < 0.001). SVV was significantly increased at the time of shock (8.8 ± 1.2% at BL vs. 22.7 ± 2.7%, *P* = 0.001, Fig. 4A). Hb significantly decreased after initial resuscitation (S + 1 h Hb 9.58 ± 0.35 g/dL, *P* < 0.001).

### Organ failure and inflammation

**Fig. 5:**
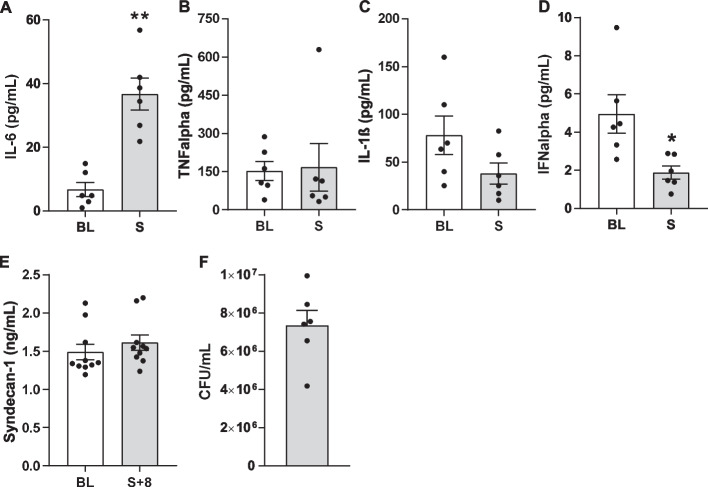
Immunological response to the septic inoculum by time of clinical occurrence of septic shock and marker of glycocalyx damage. **A** Evidence of a significant increase in proinflammatory IL-6 at the time of shock (BL: 6.74 ± 2.23 pg/mL, S: 36.76 ± 5.03 pg/mL). *n* = 6, paired *t* test. **B** TNFalpha level did not increase by clinical occurrence of septic shock *n* = 6, paired *t* test. **C** IL-1ß also did not increase by clinical onset of septic shock, *n* = 6, paired *t* test. **D** Significant decrease of IFNalpha levels (BL: 4.95 ± 1.00 pg/mL to S: 1.88 ± 0.35 pg/mL), *n* = 6, paired *t* test. **E** Syndecan-1 level did not significantly increase from 1.49 ± 0.1 ng/mL (BL) to S + 8 to the end of the experiment (S + 8, 1.61 ± 0.1 ng/mL), *n* = 10, paired *t* test, mean ± SEM. **F** Microbiological analyse of the faecal inoculum with determination of CFU/mL. *n* = 6. *P < 0.01, **P < 0.001. BL = baseline, S = shock. mean ± SEM

A significant decrease in urine output (2.4 ± 0.39 to 0.18 ± 0.56 mL/kg, *P* = 0.004) indicated acute kidney injury (AKI) and was accompanied by significant hyperkalaemia (4.7 ± 0.14 to 6.1 ± 0.16 mmol/L, *P* = 0.002) and a significant increase in creatinine from BL (1.28 ± 0.08 mg/dL) to shock time (2.06 ± 0.09 mg/dL; *P* < 0.001). Urine output increased after initial resuscitation (Fig. 4B–D). In line with sepsis-induced kidney injury, acute tubular injury was detected in more than 50% of the tubules with a significant increase compared to controls post mortem (score 0.4 ± 0.2 to 3.4 ± 0.3, *P* = 0.001). Impaired glomerular integrity appeared in 25–50% of the glomeruli (score 0.2 ± 0.2 to 2.5 ± 0.5, *P* = 0.01) (Fig. 4K, L). In addition, there was a significant increase in body temperature from baseline (39.2 ± 0.06 °C) until the onset of septic shock (40.9 ± 0.16 °C (*P* < 0.001)). The ventilatory and oxygen dynamic parameters showed no significant alteration. Arterial oxygen saturation (SaO_2_) was consistently above 95% in all animals during the experiment, verified by arterial BGA. Arterial oxygen partial pressure (PaO_2_) as well as central venous oxygen partial pressure (PzvO_2_) showed no statistically significant changes, while the inspiratory oxygen fraction (30% ± 5%) remained constant. Arterial carbon dioxide partial pressure (PaCO_2_) was kept constant at a mean of 44.6 + 0.5 mmHg. Central venous saturation (ScvO_2_) remained above a mean of 74% at all times and neither ScvO_2_ nor central venous carbon dioxide partial pressure (PcvCO_2_) showed significant changes (Table 2). Inflammation occurred with a significant increase in serum interleukin-6 (IL-6) level from a mean of 6.74 ± 2.23 pg/mL at baseline to shock with a mean of 36.76 ± 5.03 pg/mL (*P* = 0.005, Fig. 5A). The other mainly proinflammatory cytokines such as IL-1ß and TNFalpha showed no relevant changes between the two measure points (Fig. 5B, C). Interferon alpha (IFNalpha) showed a significant decrease by onset of septic shock (BL 4.95 ± 1.00 pg/mL to S 1.88 ± 0.35 pg/mL, *P* = 0.019) (Fig. 5D). The other exanimated parameters showed no relevant change. Syndecan-1 concentration showed no significant change comparing mean values at the beginning (1.49 ± 0.1 ng/mL) and at the end of the experiment (1.61 ± 0.1 ng/mL) (Fig. 5E).

**Table 2:** Arterial and venous oxygen dynamics changes during the experiment

	BL	S	S + 1	S + 2	S + 3	S + 4	S + 5	S + 6	S + 7	S + 8
SaO_2_ (%)	99.7 ± 0.0	99.5 ± 0.1	99.3 ± 0.2	99.2 ± 0.4	99.4 ± 0.2	99.2 ± 0.3	99.1 ± 0.2	99.3 ± 0.2	98.4 ± 1.2	99.3 ± 0.2
PaO_2_ (mmHg)	218.6 ± 8.3	197.4 ± 10.1	179.8 ± 9.3	185.5 ± 11.1	172.7 ± 9.9	179.2 ± 11.6	170.0 ± 70.6	181.5 ± 11.5	178.1 ± 12.9	177.5 ± 8.5
PaCO_2_ (mmHg)	42.6 ± 0.9	47.6 ± 1.5	45.8 ± 1.0	43.2 ± 2.3	44.6 ± 1.4	43.6 ± 1.4	46.1 ± 1.2	43.5 ± 1.6	44.4 ± 1.9	44.8 ± 1.3
pH art	7.46 ± 0.01	7.35 ± 0.02	7.38 ± 0.01	7.38 ± 0.01	7.38 ± 0.02	7.40 ± 0.02	7.38 ± 0.01	7.39 ± 0.01	7.39 ± 0.02	7.38 ± 0.02
ScvO_2_ (%)	80.5 ± 3.0	74.2 ± 3.3	86.5 ± 1.4	87.7 ± 1.6	86.9 ± 1.1	86.4 ± 1.6	88.5 ± 1.1	89.0 ± 0.9	89.4 ± 1.0	88.0 ± 0.9
PcvO_2_ (mmHg)	56.6 ± 3.3	68.5 ± 11.5	71.0 ± 3.8	72.3 ± 4.3	69.4 ± 2.5	69.1 ± 3.2	71.7 ± 2.6	74.8 ± 2.8	75.9 ± 3.9	71.4 ± 2.3
PcvCO_2_ (mmHg)	51.2 ± 1.7	64.9 ± 1.7	54.8 ± 2.1	51.7 ± 1.5	52.7 ± 1.2	51.2 ± 1.6	50.2 ± 1.3	50.4 ± 1.4	50.1 ± 1.3	53.6 ± 2.3
pH ven	7.40 ± 0.01	7.23 ± 0.02	7.33 ± 0.02	7.34 ± 0.01	7.34 ± 0.01	7.35 ± 0.01	7.36 ± 0.01	7.35 ± 0.01	7.36 ± 0.01	7.34 ± 0.02

Data are presented as mean ± SEM. BL = baseline, S = shock

### Microbial inoculum

The microbiological analysis showed a mean total bacterial count of 7.35*10^6^ ± 5.60*10^5^/mL which corresponded to an applied amount of 1.47*10^9^ CFU per pig. The samples differed in their total CFU/mL from 4.14*10^6^ ± 5.87*10^5^/mL to 9.95*10^6^ ± 1.59*10^6^/mL (Fig. 5F). The identified pathogens are listed in Table 3. The lead germ in the gram-positive range was enterococcus hirae and in the gram-negative range escherichia coli.

**Table 3:** Microbiological analysis of the faecal inoculum

Identified pathogens (MALDI)
Alkalihalobacillus clausii
Bacillus spp.
– Bacillus licheniformis
– Bacillus pumilus
– Bacilluzs cereus
Cytobacillus kochii
Enterococcus hirae
Escherichia coli
Staphylococcus spp.
– Staphylococcus arlettae
– Staphylococcus sciuri
– Staphylococcus xylosus
Streptococcus spp.
– Streptococcus alactolyticus
– Streptococcus hyointestinalis

## Discussion

Until today, most therapeutic approaches for sepsis and septic shock that were promising in preclinical models and evolved into a phase 3 clinical trial failed to proof effective for the treatment of sepsis in humans. Even worse, some therapies that were promising in animal models worsened outcome of septic patients, for example, TNF alpha blockers, that showed promising preclinical results but were harmful in patients [11]. Given the failure of the investigated agents, there are several considerations regarding the lack of transferability of animal models to ICUs and their limitations. For example, animal models exhibit great heterogeneity depending on species, septic inoculum, and resuscitation therapy, making them difficult to compare [9, 22]. Translational failure is thus a major obstacle and a constant challenge for preclinical research. Therefore, to address this challenge and thus translational failure, animal models orientated on human disease characteristics are needed.

In this regard, the minimum requirements for a good animal model of sepsis have recently been summarized by the International Expert Consensus for Preclinical Sepsis Studies (MQiTPSS [27]) and critically discuss major points such as study design requirements and clear requirements for the clinical relevance of the animal model, such as a physiological focus of infection and describing an organ dysfunction. In addition, animal models should also experience therapeutic standards comparable to humans, such as fluid therapy with fluid resuscitation and individualized fluid therapy, as well as antimicrobial therapy. However, therapeutic interventions should take place after implementation of the septic insult mimicking clinical care [27]. Few animal models meet these requirements; most recently, Rutai et al. [32] implemented many aspects such as differentiated fluid therapy in their porcine sepsis model, but did not provide antimicrobial therapy, for example. Furthermore, it should be noted that in this work there was a high heterogeneity of the study group due to the clinical response to the septic stimulus. In their investigation, 8 of 27 pigs had to be excluded because of an inadequate septic response - 5 animals did not respond to sepsis induction and 3 overreacted. Although all animals were subjected to the same treatment, the study group had to be further divided into two groups according to the clinical occurrence of sepsis or septic shock.

Our aim was to develop a clinically relevant large animal model of sepsis with septic shock in accordance with MQiTPSS and the third international consensus definition (Sepsis-3) in which resuscitation is approximately guideline conform with fluid therapy based on requirement, antimicrobial and vasopressor therapy [10, 27]. In our study, however, we observed septic shock with typical hemodynamic response in all 10 animals. After induction of faecal peritonitis, shock developed after 4.8 ± 0.29 h including acute organ injury and resulted in elevated serum lactate levels. To ensure a definite and severe arterial hypotension in response to infection, we defined onset of septic shock by reaching MAP < 60 mmHg (instead of 65 mmHg). Expanded hemodynamic monitoring using transpulmonary thermodilution is a standard in the management of septic shock patients in the ICU to optimize hemodynamic therapy. Previous studies have shown that thermodilution is also a reliable option in experimental preclinical settings of animal models [15, 33]. The present model with initial hypodynamic shock at onset and hyperdynamic circulation after initial resuscitation mimics the typical phenotype of human septic shock. In observing the development of acute kidney injury, our model also meets the Sepsis-3 definition, which describes sepsis as a life-threatening organ dysfunction caused by a dysregulated host response to infection [34]. To achieve the goal of increased serum lactate, we needed a high amount of faeces, incubated overnight, to ensure shock onset in all pigs. Given the comparability with the clinical diagnosis of sepsis and septic shock in humans, we hope that this model will lead to increased comparability of animal models according to the MQiTPSS in the exploration of new therapeutic options for sepsis and septic shock.

In addition to the macrohemodynamic response of the animals in septic shock, we also quantified a part of the immunologic response. The dysregulated host immune response underlying sepsis plays an important role in sepsis-related mortality by exacerbating organ damage. Cytokines are released during infection and play an important role in cell activation and signaling. Macrophages, monocytes and other non-immune cells are producing interleukin-6 (IL-6) in acute inflammation. IL-6 is part of the cytokine storm in inflammation and high IL-6 levels are associated with worse outcome and high mortality [5, 16]. Our model showed a significant increase of IL-6 levels by the time of clinical occurrence of septic shock. IL-6 may also compromise the vascular barrier integrity and lead to vascular leakage, which was observed by haemoconcentration, significantly increased SVV and large fluid requirements [16]. Other studies showed a late increase in IL-6 and IL-1ß and an early increase in TNF-alpha in comparison [3, 28]. Thus, the absence of a significant increase in other mainly proinflammatory cytokines, such as IL-1ß or TNF-alpha, with a concomitant increase in IL-6 in our model could be due to the timing of blood collection. Type I interferons are also mainly proinflammatory, and there is evidence that IFN-alpha can cause endothelial damage [6]. We observed a drop in serum IFN-alpha levels which may be explainable due to its primary release in viral infections, as seen in porcine viral infection models [25]. As increasing evidence points towards the importance of endothelial integrity for sepsis outcome, we also analyzed serum levels of syndecan-1 at the beginning and 8 h after septic shock as a marker for endothelial glycocalyx degradation. Comparing these two timepoints, we observed no difference in syndecan-1 levels, which may be due to the fact that pigs were kept euvolemic throughout the experiment using hemodynamic monitoring [2, 36]. Thus, syndecan-1 may be reliable as a marker of endothelial damage in humans but not in the euvolemic pig.

Different limitations apply. We used young and healthy female pigs without any comorbidities, which makes it difficult to compare with the usually elderly patients with sepsis in the ICU and may not account for the differences in sex-specific characteristics of sepsis. Consequently, the transferability to the clinical collective may be reduced. Despite the use of young healthy animals, interindividual variation in disease severity was evident as indicated, for example, by varying vasopressor requirements. We also did not study a healthy sham control group to determine the effects of surgical trauma and anaesthesia. However, we performed baseline measurements in the physiological range of healthy pigs, so that we assume that the changes in physiological parameters were caused by the infection. In the absence of other options, surgical intervention is required to induce sepsis, so the procedure is still experimental and did not emerge spontaneously. In addition, the septic focus has not been surgically addressed, as it would have been the case in clinical sepsis management.

## Conclusion

Animal models of sepsis and septic shock are widely used for research and identification of new therapeutic options. However, the translation of preclinical research results into clinical practice mostly failed. To narrow this gap between laboratory bench results and clinical bedside therapeutic approaches, we developed a porcine septic shock model that meets the clinical criteria of the Sepsis-3 definition. The present model mimics the typical human sepsis phenotype of early proinflammatory response with capillary leakage and hypovolemia followed by a hyperdynamic shock state after initial resuscitation and may thus be used to facilitate translational sepsis research relevant to human pathology.

## Supplementary Information

**Additional file 1: Table S1**. References Table 1.

## Data Availability

The data sets used and analysed during the current study are available from the corresponding author on reasonable request.

## Abbreviations

AKI: Acute kidney injury
ATI: Acute tubular injury
BL: Baseline
BW: Body weight
CFU: Colony forming units
CI: Cardiac index
CPI: Cardiac power index
DAP: Diastolic arterial pressure
ELISA: Enzyme-linked immunosorbent essay
EVLI: Extravascular lungwater index
GEDI: Global end-diastolic volume index
GEF: Global ejection fraction
Hb: Haemoglobin
HR: Heart rate
IFN: Interferon
IL: Interleukin
MALDI-TOF: Matrix assisted laser desorption ionization time of flight
MAP: Mean arterial pressure
MQiTPSS: Minimum quality threshold in pre-clinical sepsis studies
NE: Norepinephrine
PaCO_2_: Arterial carbon dioxide partial pressure
PaO_2_: Arterial oxygen partial pressure
PcvO_2_: Central venous oxygen partial pressure
PcvCO_2_: Central venous carbon dioxide partial pressure
PVPI: Pulmonary vascular permeability index
S: Shock
SAP: Systolic arterial pressure
SaO_2_: Arterial oxygen saturation
ScvO_2_: Central venous oxygen saturation
SVRI: System vascular resistance index
SVV: Stroke volume variation
TNF alpha: Tumor necrosis factor alpha
