# Supplementary material for: A model of porcine polymicrobial septic shock
Source: Intensive Care Med Exp. 2023 Jun 2;11:31. doi: 10.1186/s40635-023-00513-7 (PMC10235002; doi:10.1186/s40635-023-00513-7)
Supplement: Supplementary file 1 — Additional file 1: Table S1. References Table 1. [file 40635_2023_513_MOESM1_ESM.docx]

**Table I:** References table 1.

Al-Obeidallah, M., Jarkovská, D., Valešová, L., Horák, J., Jedlička, J., Nalos, L., Chvojka, J., Švíglerová, J., Kuncová, J., and Beneš, J., et al. (2021). SOFA Score, Hemodynamics and Body Temperature Allow Early Discrimination between Porcine Peritonitis-Induced Sepsis and Peritonitis-Induced Septic Shock. Journal of personalized medicine *11*. https://doi.org/10.3390/jpm11030164.

Azevedo, L.C.P. de, Park, M., Noritomi, D.T., Maciel, A.T., Brunialti, M.K., and Salomão, R. (2007). Characterization of an animal model of severe sepsis associated with respiratory dysfunction. Clinics (Sao Paulo, Brazil) *62*, 491-498. https://doi.org/10.1590/s1807-59322007000400017.

Barth, E., Bassi, G., Maybauer, D.M., Simon, F., Gröger, M., Oter, S., Speit, G., Nguyen, C.D., Hasel, C., and Möller, P., et al. (2008). Effects of ventilation with 100% oxygen during early hyperdynamic porcine fecal peritonitis. Critical Care Medicine *36*, 495-503. https://doi.org/10.1097/01.CCM.0B013E318161FC45.

Bollen Pinto, B., Ferrario, M., Herpain, A., Brunelli, L., Bendjelid, K., Carrara, M., and Pastorelli, R. (2022). Metabolites Concentration in Plasma and Heart Tissue in Relation to High Sensitive Cardiac Troponin T Level in Septic Shock Pigs. Metabolites *12*. https://doi.org/10.3390/metabo12040319.

Carrara, M., Herpain, A., Baselli, G., and Ferrario, M. (2019). A Mathematical Model of dP/dt Max for the Evaluation of the Dynamic Control of Heart Contractility in Septic Shock. IEEE transactions on bio-medical engineering *66*, 2719-2727. https://doi.org/10.1109/TBME.2019.2894333.

Chvojka, J., Martinkova, V., Benes, J., Valesova, L., Danihel, V., Nalos, L., and Matejovic, M. (2020). Mechanical Circulatory Support in Refractory Vasodilatory Septic Shock: a Randomized Controlled Porcine Study. Shock (Augusta, Ga.) *53*, 124-131. https://doi.org/10.1097/SHK.0000000000001329.

Corrêa, T.D., Jeger, V., Pereira, A.J., Takala, J., Djafarzadeh, S., and Jakob, S.M. (2014). Angiotensin II in septic shock: effects on tissue perfusion, organ function, and mitochondrial respiration in a porcine model of fecal peritonitis. Critical Care Medicine *42*, e550-9. https://doi.org/10.1097/CCM.0000000000000397.

Corrêa, T.D., Pereira, A.J., Brandt, S., Vuda, M., Djafarzadeh, S., Takala, J., and Jakob, S.M. (2017). Time course of blood lactate levels, inflammation, and mitochondrial function in experimental sepsis. Critical care (London, England) *21*, 105. https://doi.org/10.1186/s13054-017-1691-4.

Derive, M., Boufenzer, A., Bouazza, Y., Groubatch, F., Alauzet, C., Barraud, D., Lozniewski, A., Leroy, P., Tran, N., and Gibot, S. (2013). Effects of a TREM-like transcript 1-derived peptide during hypodynamic septic shock in pigs. Shock (Augusta, Ga.) *39*, 176-182. https://doi.org/10.1097/SHK.0b013e31827bcdfb.

Ferrario, M., Brunelli, L., Su, F., Herpain, A., and Pastorelli, R. (2019). The Systemic Alterations of Lipids, Alanine-Glucose Cycle and Inter-Organ Amino Acid Metabolism in Swine Model Confirms the Role of Liver in Early Phase of Septic Shock. Frontiers in physiology *10*, 11. https://doi.org/10.3389/fphys.2019.00011.

Garcia, B., Su, F., Dewachter, L., Favory, R., Khaldi, A., Moiroux-Sahraoui, A., Annoni, F., Vasques-Nóvoa, F., Rocha-Oliveira, E., and Roncon-Albuquerque, R., et al. (2022). Myocardial effects of angiotensin II compared to norepinephrine in an animal model of septic shock. Critical care (London, England) *26*, 281. https://doi.org/10.1186/s13054-022-04161-3.

Hauser, B., Barth, E., Bassi, G., Simon, F., Gröger, M., Oter, S., Speit, G., Ploner, F., Möller, P., and Wachter, U., et al. (2009). Hemodynamic, metabolic, and organ function effects of pure oxygen ventilation during established fecal peritonitis-induced septic shock. Critical Care Medicine *37*, 2465-2469. https://doi.org/10.1097/CCM.0b013e3181aee8ad.

Hiltebrand, L.B., Krejci, V., Jakob, S.M., Takala, J., and Sigurdsson, G.H. (2007). Effects of vasopressin on microcirculatory blood flow in the gastrointestinal tract in anesthetized pigs in septic shock. Anesthesiology *106*, 1156-1167. https://doi.org/10.1097/01.anes.0000267599.02140.86.

Hiltebrand, L.B., Krejci, V., and Sigurdsson, G.H. (2004). Effects of dopamine, dobutamine, and dopexamine on microcirculatory blood flow in the gastrointestinal tract during sepsis and anesthesia. Anesthesiology *100*, 1188-1197. https://doi.org/10.1097/00000542-200405000-00022.

Hiltebrand, L.B., Krejci, V., tenHoevel, M.E., Banic, A., and Sigurdsson, G.H. (2003). Redistribution of microcirculatory blood flow within the intestinal wall during sepsis and general anesthesia. Anesthesiology *98*, 658-669. https://doi.org/10.1097/00000542-200303000-00014.

Horak, J., Nalos, L., Martinkova, V., Tegl, V., Vistejnova, L., Kuncova, J., Kohoutova, M., Jarkovska, D., Dolejsova, M., and Benes, J., et al. (2020). Evaluation of Mesenchymal Stem Cell Therapy for Sepsis: A Randomized Controlled Porcine Study. Frontiers in immunology *11*, 126. https://doi.org/10.3389/fimmu.2020.00126.

Jarkovska, D., Markova, M., Horak, J., Nalos, L., Benes, J., Al-Obeidallah, M., Tuma, Z., Sviglerova, J., Kuncova, J., and Matejovic, M., et al. (2018). Cellular Mechanisms of Myocardial Depression in Porcine Septic Shock. Frontiers in physiology *9*, 726. https://doi.org/10.3389/fphys.2018.00726.

Jarkovska, D., Valesova, L., Chvojka, J., Benes, J., Sviglerova, J., Florova, B., Nalos, L., Matejovic, M., and Stengl, M. (2015). Heart Rate Variability in Porcine Progressive Peritonitis-Induced Sepsis. Frontiers in physiology *6*, 412. https://doi.org/10.3389/fphys.2015.00412.

Ji, M.-H., Yang, J.-J., Wu, J., Li, R.-Q., Li, G.-M., Fan, Y.-X., and Li, W.-Y. (2012). Experimental sepsis in pigs--effects of vasopressin on renal, hepatic, and intestinal dysfunction. Upsala journal of medical sciences *117*, 257-263. https://doi.org/10.3109/03009734.2011.650796.

Kieslichova, E., Rocen, M., Merta, D., Kudla, M., Splichal, I., Cap, J., Viklicky, O., and Gürlich, R. (2013). The effect of immunosuppression on manifestations of sepsis in an animal model of cecal ligation and puncture. Transplantation Proceedings *45*, 770-777. https://doi.org/10.1016/j.transproceed.2012.07.159.

Krejci, V., Hiltebrand, L.B., Erni, D., and Sigurdsson, G.H. (2003). Endothelin receptor antagonist bosentan improves microcirculatory blood flow in splanchnic organs in septic shock. Critical Care Medicine *31*, 203-210. https://doi.org/10.1097/00003246-200301000-00031.

Krejci, V., Hiltebrand, L.B., Jakob, S.M., Takala, J., and Sigurdsson, G.H. (2007). Vasopressin in septic shock: effects on pancreatic, renal, and hepatic blood flow. Critical care (London, England) *11*, R129. https://doi.org/10.1186/cc6197.

Krejci, V., Hiltebrand, L.B., and Sigurdsson, G.H. (2006). Effects of epinephrine, norepinephrine, and phenylephrine on microcirculatory blood flow in the gastrointestinal tract in sepsis. Critical Care Medicine *34*, 1456-1463. https://doi.org/10.1097/01.CCM.0000215834.48023.57.

Kuncová, J., Sýkora, R., Chvojka, J., Svíglerová, J., Stengl, M., Kroužecký, A., Nalos, L., and Matějovič, M. (2011). Plasma and tissue levels of neuropeptide y in experimental septic shock: relation to hemodynamics, inflammation, oxidative stress, and hemofiltration. Artificial organs *35*, 625-633. https://doi.org/10.1111/j.1525-1594.2010.01154.x.

Kurtz, P., d'Avila, J.C., Prado, D., Madeira, C., Vargas-Lopes, C., Panizzutti, R., Azevedo, L.C.P., and Bozza, F.A. (2019). Cerebral Multimodal Monitoring in Sepsis: An Experimental Study. Shock (Augusta, Ga.) *51*, 228-234. https://doi.org/10.1097/SHK.0000000000001138.

Laroye, C., Lemarié, J., Boufenzer, A., Labroca, P., Cunat, L., Alauzet, C., Groubatch, F., Cailac, C., Jolly, L., and Bensoussan, D., et al. (2018). Clinical-grade mesenchymal stem cells derived from umbilical cord improve septic shock in pigs. Intensive care medicine experimental *6*, 24. https://doi.org/10.1186/s40635-018-0194-1.

Li, C., Zhang, P., Cheng, X., and Chen, J. (2013). High-volume hemofiltration reduces the expression of myocardial tumor necrosis factor-alpha in septic shock pigs. Artificial organs *37*, 196-202. https://doi.org/10.1111/j.1525-1594.2012.01536.x.

Marx, G., Cobas Meyer, M., Schuerholz, T., Vangerow, B., Gratz, K.F., Hecker, H., Sümpelmann, R., Rueckoldt, H., and Leuwer, M. (2002). Hydroxyethyl starch and modified fluid gelatin maintain plasma volume in a porcine model of septic shock with capillary leakage. Intensive Care Med *28*, 629-635. https://doi.org/10.1007/s00134-002-1260-3.

Marx, G., Pedder, S., Smith, L., Swaraj, S., Grime, S., Stockdale, H., and Leuwer, M. (2004). Resuscitation from septic shock with capillary leakage: hydroxyethyl starch (130 kd), but not Ringer's solution maintains plasma volume and systemic oxygenation. Shock *21*, 336-341. https://doi.org/10.1097/00024382-200404000-00008.

Marx, G., Pedder, S., Smith, L., Swaraj, S., Grime, S., Stockdale, H., and Leuwer, M. (2006). Attenuation of capillary leakage by hydroxyethyl starch (130/0.42) in a porcine model of septic shock. Critical Care Medicine *34*, 3005-3010. https://doi.org/10.1097/01.CCM.0000242755.74063.ED.

Merz, T., Denoix, N., Wigger, D., Waller, C., Wepler, M., Vettorazzi, S., Tuckermann, J., Radermacher, P., and McCook, O. (2020). The Role of Glucocorticoid Receptor and Oxytocin Receptor in the Septic Heart in a Clinically Relevant, Resuscitated Porcine Model With Underlying Atherosclerosis. Frontiers in endocrinology *11*, 299. https://doi.org/10.3389/fendo.2020.00299.

Messerer, D.A.C., Datzmann, T., Baranowsky, A., Peschel, L., Hoffmann, A., Gröger, M., Amling, M., Wepler, M., Nussbaum, B.L., and Jiang, S., et al. (2022). Systemic calcitonin gene-related peptide receptor antagonism decreases survival in a porcine model of polymicrobial sepsis: blinded randomised controlled trial. British journal of anaesthesia *128*, 864-873. https://doi.org/10.1016/j.bja.2021.11.042.

Nußbaum, B.L., McCook, O., Hartmann, C., Matallo, J., Wepler, M., Antonucci, E., Kalbitz, M., Huber-Lang, M., Georgieff, M., and Calzia, E., et al. (2016). Left ventricular function during porcine-resuscitated septic shock with pre-existing atherosclerosis. Intensive care medicine experimental *4*, 14. https://doi.org/10.1186/s40635-016-0089-y.

Nußbaum, B.L., Vogt, J., Wachter, U., McCook, O., Wepler, M., Matallo, J., Calzia, E., Gröger, M., Georgieff, M., and Wood, M.E., et al. (2017). Metabolic, Cardiac, and Renal Effects of the Slow Hydrogen Sulfide-Releasing Molecule GYY4137 During Resuscitated Septic Shock in Swine with Pre-Existing Coronary Artery Disease. Shock (Augusta, Ga.) *48*, 175-184. https://doi.org/10.1097/SHK.0000000000000834.

Ospina-Tascón, G.A., García Marin, A.F., Echeverri, G.J., Bermudez, W.F., Madriñán-Navia, H., Valencia, J.D., Quiñones, E., Rodríguez, F., Marulanda, A., and Arango-Dávila, C.A., et al. (2017). Effects of dobutamine on intestinal microvascular blood flow heterogeneity and O2 extraction during septic shock. Journal of applied physiology (Bethesda, Md. : 1985) *122*, 1406-1417. https://doi.org/10.1152/japplphysiol.00886.2016.

Park, I., Lee, J.H., Jang, D.-H., Kim, D., Chang, H., Kwon, H., Kim, S., Kim, T.S., and Jo, Y.H. (2019). Characterization of Fecal Peritonitis-Induced Sepsis in a Porcine Model. The Journal of surgical research *244*, 492-501. https://doi.org/10.1016/j.jss.2019.06.094.

Regueira, T., Djafarzadeh, S., Brandt, S., Gorrasi, J., Borotto, E., Porta, F., Takala, J., Bracht, H., Shaw, S., and Lepper, P.M., et al. (2012). Oxygen transport and mitochondrial function in porcine septic shock, cardiogenic shock, and hypoxaemia. Acta anaesthesiologica Scandinavica *56*, 846-859. https://doi.org/10.1111/j.1399-6576.2012.02706.x.

Rosário, A.L., Park, M., Brunialti, M.K., Mendes, M., Rapozo, M., Fernandes, D., Salomão, R., Laurindo, F.R., Schettino, G.P., and Azevedo, L.C.P. (2011). SvO(2)-guided resuscitation for experimental septic shock: effects of fluid infusion and dobutamine on hemodynamics, inflammatory response, and cardiovascular oxidative stress. Shock (Augusta, Ga.) *36*, 604-612. https://doi.org/10.1097/SHK.0b013e3182336aa4.

Rutai, A., Zsikai, B., Tallósy, S.P., Érces, D., Bizánc, L., Juhász, L., Poles, M.Z., Sóki, J., Baaity, Z., and Fejes, R., et al. (2022). A Porcine Sepsis Model With Numerical Scoring for Early Prediction of Severity. Frontiers in medicine *9*, 867796. https://doi.org/10.3389/fmed.2022.867796.

Schmidt, C., Lautenschläger, C., Petzold, B., Sakr, Y., Marx, G., and Stallmach, A. (2013). Confocal laser endomicroscopy reliably detects sepsis-related and treatment-associated changes in intestinal mucosal microcirculation. British journal of anaesthesia *111*, 996-1003. https://doi.org/10.1093/bja/aet219.

Schuerholz, T., Meyer, M.C., Friedrich, L., Przemeck, M., Sümpelmann, R., and Marx, G. (2006). Reliability of continuous cardiac output determination by pulse-contour analysis in porcine septic shock. Acta anaesthesiologica Scandinavica *50*, 407-413. https://doi.org/10.1111/j.1399-6576.2006.00982.x.

Simon, F., Giudici, R., Scheuerle, A., Gröger, M., Asfar, P., Vogt, J.A., Wachter, U., Ploner, F., Georgieff, M., and Möller, P., et al. (2009). Comparison of cardiac, hepatic, and renal effects of arginine vasopressin and noradrenaline during porcine fecal peritonitis: a randomized controlled trial. Critical care (London, England) *13*, R113. https://doi.org/10.1186/cc7959.

Sykora, R., Chvojka, J., Krouzecky, A., Radej, J., Kuncova, J., Varnerova, V., Karvunidis, T., Novak, I., and Matejovic, M. (2009). Coupled plasma filtration adsorption in experimental peritonitis-induced septic shock. Shock (Augusta, Ga.) *31*, 473-480. https://doi.org/10.1097/SHK.0b013e318188dec5.

Wauters, J., Mesotten, D., van Zwam, K., van Pelt, J., Thiessen, S., Dieudonné, A.-S., Vander Borght, S., van den Berghe, G., and Wilmer, A. (2010). The impact of resuscitated fecal peritonitis on the expression of the hepatic bile salt transporters in a porcine model. Shock (Augusta, Ga.) *34*, 508-516. https://doi.org/10.1097/SHK.0b013e3181dfc4b4.
